# Primary Myoma of the Broad Ligament

**Published:** 1888-02

**Authors:** Bayard Holmes

**Affiliations:** 1535 Bowen Avenue


### The Medical Journal and Examiner.

VOLUME LVI. FEBRUARY, 1888. NUMBER 2.

### REPORT OF A CASE OF PRIMARY MYOMA OF THE BROAD LIGAMENT, AND A TABLE OF SEVENTEEN COLLECTED CASES.

BY DR. BAYARD HOLMES.

[Read before the Gynæcological Society of Chicago.]

I have the pleasure of presenting a specimen of a fibroid tumor, resembling those of the uterus, and calling your attention to a small group of neoplasms termed by Sänger "primary desmoid tumors of the broad ligament."

1. The patient from whom this specimen was removed was a well-developed woman, 35 years old. She had been twice married. By the first marriage she was without issue. About a year after the second marriage she gave birth to a child that is now three years old. At this confinement there was a laceration of the cervix uteri, which was operated upon in February, 1886. The pelvis was thoroughly examined at that time, while the patient was under the anæsthetic, and nothing abnormal detected. Following the operation there was a slight retroversion, which was treated with a pessary for a month or two. Toward the last of May, 1886, this pessary was removed by the patient herself. The menstruation was scanty in June, and she consulted her physician, who concluded from an increased size of the uterus that it was pregnant, and refrained from further examination. In July again the menstruation was normal, and an examination at that time first discovered a small tumor, about the size of a black walnut, just behind the uterus. The patient was put in the knee-chest position and the tumor pushed back almost out of reach. It was movable, and free from the uterus. The tumor had all the appearance of a displaced, enlarged ovary, and it was so considered at the time. Repeated examinations during the following months revealed its rather rapid growth and its increasing immovability. A dull fluctuation was repeatedly detected. At last the inconvenience to the patient became so great that she decided for herself that it should be removed.

The patient had never had any pelvic inflammation, and the puerperium was unattended by sepsis.

I had the privilege of making the necessary antiseptic preparation antecedent to the operation. Every scientifically indicated preliminary precaution in the preparation of the room, the instruments, the patient, and the operators, was taken. The room was large and airy, having three south windows and one opening to the west. The house was a frame dwelling which had been in use many years. The floors were carpeted throughout the house. I had the carpet removed from the operating room, which was on the second floor, all the woodwork thoroughly scrubbed, and the walls freshly papered. There was not sufficient time to lay a new floor, and the old one was too much worn to be made clean. I had it covered with a carpet of thick, white duck. After this had been tacked down, all the woodwork and the necessary furniture and this carpet were scrubbed with a 1:1000 sublimate solution. Just before the operation the temperature was raised to 90° F., and a pound of carbolic acid evaporated in the room.

The operation was performed on the morning of the 5th of June, 1887. There were present the operator, Dr. J. W. Streeter, three assistants, and the nurse. Upon opening the abdomen, it was found impossible to bring the tumor in the line of the incision. It lay close to the laterally turned uterus, and occupied all the space in the right side of the pelvis. It was found impossible to move it in any direction more than an inch. Two-thirds of the tumor was visible, covered by smooth peritoneum, which was reflected backward and directly forward upon the walls of the pelvis. No ovary was found on the right side. The left ovary seemed perfectly normal. The tumor felt hard, elastic, and smooth. A transverse incision was made through its peritoneal covering, and the tumor carefully enucleated with dull instruments. There was very little hæmorrhage. The broad base from which the tumor was removed was then sewed through with a curved needle and strong silk. This was tied at every stitch. The peritoneal edges were trimmed off, turned in, and fastened with fine silk. The operation lasted two hours. During the following day the patient's temperature rose to 103° F., but it gradually fell to below 100° at the end of the second day. There was no other unfavorable symptom. The sutures were removed from the abdominal wound on the fourth day, and the second and last dressing applied to the abdomen. There was not a particle of suppuration at any time, nor any pain in the line of the abdominal cut, or in the region of the operation. There was just a show of blood on the vaginal tampon where it lay against the os uteri. The patient was somewhat troubled with borborygmus during the first week, and at the end of a month had an attack of gastro-enteritis. She has now regained her former health and strength, and has menstruated regularly and normally.

The tumor which I pass around has all the appearance of a uterine fibroid. It is almost perfectly spherical and smooth. It is about the size of a man's fist. The cut surface is smooth and white, with a few cavernous sinuses, filled originally with a clear, serous fluid. A piece of the fibrous capsule is still attached to one side of the tumor. The drawings which I present were made from stained sections, with a one-tenth inch immersion objective, and an inch eye-piece. The nuclei resemble very much those of unstriped muscle-fibers. There is very little, if any, pure fibrous tissue to be found in the body of the tumor. Near the blood vessels, the nuclei are ovoid or spherical, and very large. These are, no doubt, the round cells noticed by Kleinwächter (49) in small uterine fibroids. They seem to be well-nourished cells, undergoing rapid mitosis, or indirect cell division. These large, round cells are easily distinguished from tranverse sections of the long nuclei by their size and arrangement.

This tumor, then, is leiomyoma—that is, the myoma which originated from a matrix of smooth muscle tissue.

Whatever may be the cause of the development of a neoplasm, it is doubtless necessary that it should have a matrix of at least as highly specialized a grade as that presented by the tumor itself.

Such a matrix is found in the uterus, in the uterine ligaments, in the base of the ovary, in the walls of the bladder, and in the abdominal wall.

(*a*) A consideration of the history of the case shows conclusively that this tumor did not originate as a sub-peritoneal fibroid of the uterus. When first observed, it was quite a distance from the uterus, and freely movable from it in the pelvis. The pelvis was thoroughly examined under an anæsthetic four months previous to the appearance of the tumor and nothing found.

It does not seem possible that a fibroid of the uterus could be so completely displaced either by puerperal contraction, or by the traumatism it suffered at the operation in February.

There remain to be considered the possibilities of an ovarian or a ligamental origin for our tumor.

(*b*) We have four methods of demonstrating the presence of smooth muscle-fibres in the broad ligament—the embryological, the anatomical, the physiological, and the pathological. The uterine ligaments contain plain muscle-fibers, which are prolongations of the superficial embryonal layer of the uterus. These fibers extend through the ring into the mons veneris, and out on the abdominal wall to the *linea alba*, and up over the posterior wall of the bladder.

Anatomically, these smooth muscular fibers have been found and described by Rouget (14), Klebs (16), Henle (17), and Luschka (19). They are also mentioned in Quain's Anatomy.

They have also been assigned an important role in initiating labor (18). In examining the wall of cysts of the broad ligament, Spiegelberg (20) and Lawson Tait (21) have seen layers of unstriped muscular fibers three or four millimeters long. Other investigators have sometimes found and sometimes missed these bundles in the walls of parovarian cysts. There is, then, no doubt that a suitable matrix for the development of leiomyomas is found in the broad ligament.

Graetzer (26), investigating the origin of the tumors of the abdominal wall, has opened up all the possibilities of the origin of fibroid neoplasms in every part of the body. According to the theory of Cohnheim, there must be a matrix derived from the mesoblastic layer of the embryo. Thus the fibroids of the skin arise from *musculi erectores pili;* those of the æsophagus, stomach, intestinal canal, and prostate, from the plain muscular fibers of these organs, and those of the abdominal wall, from fibers going out from the uterus through the ligaments in the direction of their embryonal attachment.

Twelve well diagnosticated connective-tissue tumors of the round ligament have been collected by Sänger (2). These represent almost every possible theoretical position in which such a neoplasm could be found, namely, in the round ligament, in the pelvis, in the canal and outside the canal, in the walls of the abdomen, or in the *labia majora*.

Small fibroids have been occasionally found in the broad ligament. Virchow (9) has made such an observation. The tumor was as large as a bean, and so far removed from the uterus and from the ovary that no relation of origin to them could be considered. Such tumors have been thought by many investigators to arise from a displaced fragment of the ovary, or an accessory ovary.

If any doubt remained of the possibility of the primary origin of fibroid, or better, of desmoid, tumors in the broad ligament, the careful consideration of Cohnheim's post-mortem examination, reported by Freund (28), would remove it. He found on the posterior wall of the uterus and of the bladder, and between the leaves of the broad ligament, even to the outer end of it, numerous isolated myomas of all sizes, from a cherry to an apple.

Sänger (1 and 2) has collected eleven cases of primary desmoid tumors of the broad ligament, which reached a considerable size. There are several observations by other authors which swell the number of observed cases to nineteen or twenty.

Then it is both possible and probable that our tumor arose originally from the broad ligament in which it was found. It is still necessary to show that it did not arise from the ovary.

(*c*) There are, at least, three reasons for thinking that it did not arise from the ovary, namely, the form of the tumor, the microscopical elements of the tumor, and its manner of attachment.

The fibroids of the ovary are simply hypertrophies of the connective tissue of the ovary, and therefore their contour is irregular and nodulated, and not smooth and spherical like ours.

Fibroids of the ovary are composed almost entirely of fibrous tissues, and unstriped muscle-fiber is rarely and sparingly found, while our tumor is composed almost, if not entirely, of plain muscle-fibers.

Fibroids of the ovary have been shown by Leopold (28) to be connected with the ligament by a long, simple pedicle, which does not include the tube. Our tumor lay deep and sessile in the pelvis.

It is not significant that a diagnosis of an ovarian tumor was made before the operation, as this has been repeatedly done by other operators in the case of solid tumors and cysts of the ligaments.

Neither does it matter that the right ovary was not recognized, as it is frequently melted down on the surface of large cysts and becomes almost unrecognizable through pressure atrophy.

I conclude after all these considerations that this is a true primary leiomyoma of the broad ligament.

A table is appended to this article, giving a synopsis of the eleven cases collected by Sänger (1 and 2), to which I have added six other cases. Three of these are from Freund (28), one is from Bantock, and one is from an unpublished case of Fenger, and the other is the case I have just described.

In only six cases was the true position of the tumor recognized before the operation, namely, Schmid's, Miculicz's, Freund's three cases, and Fenger's.

Cystic, cavernous, and sarcomatous myomas, as well as simple myomas, are represented.

The average age of the patients is about thirty-two years, the extreme being nineteen and forty-two.

It is probable that these tumors are not as rare as this limited number would indicate, but that in the advanced stage of growth in which they come to operation, the ovary becomes drawn out and lost on the wall of the tumor, which then seems to have arisen from the lost ovary. An earlier examination would perhaps have revealed their true position.

1535 Bowen Avenue.

### LITERATURE ON TUMORS OF THE BROAD LIGAMENT.

1. SÄNGER. Ueber primäre desmoid Geschwülste der Ligamenta lata. Arch. f. Gyn., xvi, 258.

2. SÄNGER. Weitere Beiträge zur Lehre von den desmoiden Geschwülsten der Gebärmutterbänder, etc. Arch. f. Gyn., xxi, p. 279.

3. KLOB. Path. Anat. der weiblichen Sexualorgane. 1864.

4. KIEWISCH. Klin. Vortr. u. spec. Path. u. Ther. des weibl. Geschlechtsorg. 1849.

5. SCANZONI. Lehre der Krankh. der weibl. Sexualorgane. 1875.

6. SCHETELIG. Beiträge zur Diagnostic der chronischen Unterleibsgeschwlüste. Arch. f. Gyn., Bd. i, 425.

7. SCHROEDER. Lehrbuch der Frauenkrankheiten. 1879 and 1886.

8. STEIN. Inaugural dissertation. Berlin, 1876.

9. VIRCHOW. Geschwülste. Bd. iii, 1, 221, etc.

10. BANDL. Krankheiten der Tuben, der Lig., u. s. w., p.
Handbuch der Frauenkrankheiten, von BILLROTH.

11. SCHMID. Prager med. Wochenschr., 1878. 35.

12. MICULICZ. Extirpationen der Geschwülste des Uterus u. der Ligamenta lata durch die Laparotomie. Wiener med. Wochenschr., 1878, 19, 20, 21.

13. GAYET. Extirpation d'un Kyste du Ligam. large de la Matrice. Lyon Medical, 1874, No. 9, p. 542.

14. ROUGET. Recherches sur le Type des Organes Génitaux et de leurs Appareils Musculaires. Thése de Paris, 1885, No. 294.

15. HAYES and SAVAGE. Lancet, May 30th, 1874.

16. KLEBS. Handbuch der Path. Anat., iv, 841 and 839.

17. HENLE. Handbuch der Eingeweidelehre des Menschen, 1873.

18. ERBSTEIN. Arch. f. mikroskopische Anat., 2, 1866, 530.

19. LUSCHKA. Anatomie des Menschen, etc. 11, 2, 363.

20. SPIEGELBERG. Arch. f. Gyn., Bd. i.

21. LAWSON TAIT. Diseases of the Ovary. N. Y., 1883, p. 166.

22. SCANZONI. Beiträge, v, 163.

23. WINCKEL. Die Path. der weibl. Sexualorg. Leipzig, 1881, p. 455.

24. GUSSEROW. Arch. f. Gyn., x, 185.

25 FISCHEL. Ueber Parovarialcysten und parovarielle Kystome. Arch. f. Gyn., 203–210.

26. GRAETZER. Die bindgewebigen Neubildungen der Bauchwand. Inaugural dissertation. Breslau, 1879.

27. GUERIN. Sur la Structure des Ligaments larges. Acad. des Sciences, 1879.

28. FREUND, A. W. Gyn. Klinik. i, 1885, pp. 289–293.

29. KLEINWÄCHTER. Zur Entwickelung der Myome des Uterus. Zeitschr. f. Geburtsh. u. Gyn., Bd. ix, p. 68.

30. LEOPOLD. Die soliden Eierstocksgeschwülste. Arch. f. Gyn., vi, 235.

TABLE OF SEVENTEEN COLLECTED CASES.

| Observer. | Age of Patient. | Tumor recognized. | Symptoms. | Fluctuation. | Diagnosis before the operation. |
|---|---|---|---|---|---|
| 1. Schetelig. Arch. f. Gyn., I., 259. | 41 years. Multipara. | 2 years................... | Tumor elastic. In general form spherical; in places irregular and hard. Above the symphysis a tympanitic zone. Uterus movable and normally located. | Fluctuation indubitable. | Ovarian cyst. Diagnosticated by another surgeon. |
| 2. Schmid. Prager med. Wochens.,1878, No. 35, 260. | 35 years. One Child. | 1¼ years after delivery. | After previous peritonitic symptoms, rapid development in the right pelvic cavity of a movable tumor. Decendens uteri. Prolapse of the anterior vaginal wall. | Wanting.... | Solid tumor of the right broad ligament. By internal examination both ovaries were recognized. |
| 3. Miculicz. Wien. med. Wochens., 1879, No. 21, 261. | ............ | ........................... | Periodical pain in the pelvis. For two or three years ascites present. Umbilical hernia since one year. Tumor freely movable, somewhat uneven, as large as a man's head. | Wanting ... | Solid tumor of the broad ligament. |
| 4. Gayet. Lyon Medical, 1874, No. 9, p. 273. | 42 years. Multipara. | 7 years.................... | All the appearance of a simple ovarian cyst. | Present. Very pronounced. | Ovarian cyst with short pedicle. |
| 5. Sänger. Arch. f. Gyn., XVI., 284. | 19 years. One Child. | 10 months.............. | Failure of the menses to appear for seven months. Patient considered herself pregnant. Prolapse of uterus and vagina. | Present. Very pronounced. | Left side ovarian cyst . |
| 6. Bardenheuer. Drainirung d. Peritonealhoehle. Stuttgart, 1881, S. 113-118 (in Hegar). | ............ | .................... ......... | A large tumor which was located deep in the pelvis and extended somewhat above it. | ............ | ......................... |
| 7. Péan. (in Hegar). | ............ | ........................... | .... ....................... | ....... ..... | .......... ...... ..... |
| 8. Buschmann (operator, Billroth). Wiener Wochenschrift. 1880, No. 28. | 24 years 4-para. | ........................... | Increase in the circumference of the abdomen for three years. Dull elastic consistency. The percussion dullness extended to that of the liver and spleen. | ............ | .... ................. .. |
| 9. Rydygier. Deutsche Zeitschrift für Chirurgie, XV., p. 279. | Multipara.. | ........................... | ................................. | Pseudofluctuations. | Solid pediculated tumor of the uterus. |

TABLE OF SEVENTEEN COLLECTED CASES.

| Condition found at the operation. | | | Operation. | Anatomical diagnosis. | Result. | Remarks. |
|---|---|---|---|---|---|---|
| Pedicle. | Uterus. | Ovary. | | | | |
| Long, somewhat thick; attached to the right broad ligament | Normal.. | Normal ........ | .................. | Cystomyoma teleangiectodes cavernosum ligamenti lati dextri. | No operation. Death from chronic peritonitis spreading out from local suppurative peritonitis. | Uterus free without any attachment to the tumor. Left ovary normal. Right ovary drawn up on the anterior, under surface of the tumor. |
| Thin. in necrosis. | Free, infantile. | Right ovary in necrosis wholly separated from the tumor. On the left ovary a pea-sized. pedunculated fibroma. | .................. | Fibro-sarcoma of the right broad ligament. Wgt. 16 lbs. | Pedicle dropped. Course without fever. Healing by first intention. | .................... |
| Wanting. | Normal.. | Normal........ | .................... | Fibroma of the right broad ligament. Tumor strongly edematous. Wgt. 10 lbs. | Pedicle dropped. Recovery retarded by secondary intra-abdominal hæmorrhage and abdominal abscess. | On the ovarian side of the tumor, arising from the right parovarium a small dermoid cyst. |
| Short and small. | Normal.. | The left ovary behind the anterior tumor edematous, beset with follicular cysts, free from the ligmentum latum. Right ovary apparently normal. | .................... | Cystomyoma of the left broad ligament. | Attempted removal. Suturing of the residue of the cyst in the abdominal wall. Gangrene of the pelvic tissues. Death on the 14th day. | .................... |
| .......... | .......... | .............. | .................... | Pibromyoma hydropicum Ligamenti lati sinistri. Double tumor. The anterior small, intraligamentous, the large one extra ligamentous, intraperitoneal and pedunculated. Wgt. 16 lbs. | .................. | .................... |
| .......... | .......... | .............. | Laceration of the anterior wall of the rectum. Intestinal suture. Injury of a large parametrian blood vessel in arranging the drainage. | Intraligamentous myoma. | Death four hours after the operation from hæmorrhage. | .................... |
| Short .... | .......... | .............. | Pedicle treated as an ovarian pedicle. | Medullary sarcoma of the broad ligament. | Death on the second day of peritonitis. | .................... |
| None present. | Free without attachment to the tumor. | .............. | Vigorous hæmorrhage in separation from the peritoneum. The tumor had grown from the back side and turned forwards. | Retroperitoneal fibro-myoma, by which the kidney which lay in the pelvis was deformed. The sessile tumor lay between the leaves of the ligamentum latum. Wgt. 36 lbs. | Death from peritonitis on the fifth day. | The tumor was very vascular and gave the impression of a firm jelly-like tumor. It was covered with a very thin peritoneum |
| None ... | On the uterus, two subperitoneal tumors about the size of small apples. | Free .......... | The tumor was raised out of its place without the aid of instruments and without the application of a ligature. The anterior leaf of the broad ligament was grown fast to the abdominal wall. | Fibromyoma between the leaves of the right broad ligament. The tissues were here and there colloid degenerated. | Recovery.......... | .................... |

TABLE OF SEVENTEEN COLLECTED CASES.

| Observer. | Age of Patient. | Tumor recognized. | Symptoms. | Fluctuation. | Diagnosis before the operation. |
| --- | --- | --- | --- | --- | --- |
| 10. Chimiaux. Arch. de Tocol. et des mals des femmes, Juillet 1880, p. 439. | 32 years. Multipara. | ........................ | The tumor grew steadily but without pain. It appeared to arise from the uterus. It moved with its movements. Remarkable developments in the course of a half year. | ........... | Solid pediculated tumor of the uterus. |
| 11. Schroeder. Sitzung der Gesellschaft f. Geburtsh. u. Gynaek. Berlin, June 8th, 1880. Berlin Klin. Wochensch., 1881. | ............ | ........................ | ...................." ............ | ........... | .................... |
| 12. Freund; Gynaekologische Klinik, 1885, p. 289-293. | 32 years. Multipara, | Recognized 5 years, walnut sized, somewhat uneven, solid, elastic, growing more backward, separated from two smaller cherry-sized tumors by a distinct groove. Uterus movable. Both sound ovaries easily recognized. | Pain in the pelvis several weeks after her first normal labor, protracted menstruation. The tumor grew slowly, but undoubtedly. | None....... | Fibroid of the left broad ligament. |
| 13. Freund. Loc. cit. | 30 years. Nullipara. | ........................ | .... ........................ | ........... | Double fist-sized myoma of the uterus and a tumor in the left broad ligament. |
| 14. Freund. Loc. cit. | 32 yrs. Sterile wife for ten years. | 14 days after the removal of a subperitonal myoma as large as a man's fist, a plum-sized tumor of the left broad ligament was detected. | ........ ... .......... ....... | ........... | Solid tumor of the left broad ligament which became as large as a child's fist, still separated from the uterus by a distinct furrow. |
| 15. Bantock. The Brit. Gyn. Jour. X., Aug., 1887. | ............ | Tumor recognized 9 months before operation, soon after the birth of the last child. | ............ ....... ...... | ........... | Believed to be ovarian. |
| 16. Fenger. Unpublished. | 29 years. Multipara. | About one year......... | Caused little inconvenience, but aroused the suspicion of pregnancy. | None...... | Solid tumor of the left broad ligament. Separated from the ovary and uterus by a recognizable space, filling the pelvis and extending half-way to the umbilicus. Somewhat movable. Uterus and ovary separately movable. |
| 17. Myself. (Operator Streeter.) In this article. | 35 years. 1-para. | Tumor recognized 11 months before operation, when it was movable from the uterus. | .................. ......... | Pseudo-fluctuations. | Tumor of the right ovary. |

See also a report of a specimen exhibited by Dr. Constantine Goodell, Am. Journal of Obstet., Vol. X, p. 178, 1887.

TABLE OF SEVENTEEN COLLECTED CASES.

| Condition found at the operation. | | | Operation. | Anatomical diagnosis. | Result. | Remarks. |
|---|---|---|---|---|---|---|
| Pedicle. | Uterus. | Ovary. | | | | |
| Present.. | Free ..... | Free ............ | The tumor was attached to a pedicle which arose from the left broad ligament. | Cysto-sarcoma of the left broad ligament. Wgt. 12 lbs. | Death on the 13th day of peritonitis which certainly had arisen from an abscess in the neighborhood of the pedicle. | The tumor presented a trembling, soft mass, made up of wide-meshed tissues with fist-sized cavities within. No autopsy or microscopical examination of the tumor was made. |
| ........... | ........ | ............ | ............ | Fibro-myomatous tumor. | ............ | Discovery of the tumor, which was from the left broad ligament, during the removal of an eight-sided ovarian cystoma. |
| None..... | Movable.. | Both Present. Sound. | Enucleation......... | Myofibroma cysticum intra-ligamentare. Weight 13 lbs. Prof. Cohnheim confirmed the diagnosis. | After the operation, there was a secondary hæmorrhage which was controlled by compression. Trismus and tetanus on the sixth day, in spite of which patient recovered and left the hospital after six weeks. | The ureter lay bare in enucleating the sessile tumor. |
| None..... | Free ..... | Both present. | After enucleation of the myoma of the uterus, a tumor of the ligament, the size of a 5 mo. gravid uterus was removed. The peritoneal covering of the tumor was cut so that a continuous suture restored the relations of the parts to those of a normal ligament. | ............ | ............ | The left ureter lay under the side of the tumor. |
| ........... | ........ | ............ | ............ | ............ | No operation.. .... | ............ |
| None..... | Free ..... | Both present. | Sub-peritoneal enucleation. Removal of diseased right ovary. | Fibroid of the right broad ligament. The tumor arose in the outer portion of the ligament beyond the ovary which lay on its inner aspect. | Recovery........... | Peritoneum freely movable over the surface of the tumor. |
| None..... | Free ..... | Both present, healthy. | Enucleation, partial closure of the peritoneal wound. Drainage by means of a Miculicz drain introduced into the bottom of the unclosed peritoneal wound. | Fibroid of the broad ligament. Weight 4 lbs. The cut surface showed multiple nut-sized and larger fibroids melted down into one large and almost perfectly spherical tumor. | Death from sepsis on the third day. | The omentum adherent to the tumor over a considerable surface contained enormously enlarged blood-vessels (finger-sized) furnishing the principal blood supply of the tumors. |
| None..... | Free ..... | Not found...... | Transverse incision through the broad ligament and enucleation of the sessile, immovable tumor. | Leiomyoma of the right broad ligament. Wgt. 10 ounces. | Recovery without complication. In perfect condition Nov. 28, 1887. | ............ |

